# Antibacterial action mechanisms and mode of trypsin inhibitors: a systematic review

**DOI:** 10.1080/14756366.2022.2039918

**Published:** 2022-02-16

**Authors:** Amanda Maria de Souza Nascimento, Victor Hugo de Oliveira Segundo, Ana Júlia Felipe Camelo Aguiar, Grasiela Piuvezam, Thaís Souza Passos, Karla Suzanne Florentino da Silva Florentino da Silva Chaves Damasceno, Ana Heloneida de Araújo Morais

**Affiliations:** a Nutrition Postgraduate Program, Center for Health Sciences, Federal University of Rio Grande do Norte, Natal, Brazil; b Postgraduate Program in Public Health, Center for Health Sciences, Federal University of Rio Grande do Norte, Natal, Brazil; c Biochemistry and Molecular Biology Postgraduate Program, Biosciences Center, Federal University of Rio Grande do Norte, Natal, Brazil; d Department of Public Health, Center for Health Sciences, Federal University of Rio Grande do Norte, Natal, Brazil; e Department of Nutrition, Center for Health Sciences, Federal University of Rio Grande do Norte, Natal, Brazil

**Keywords:** Antimicrobial peptides, bioactive proteins, infectious diseases, bacteria

## Abstract

This systematic review (SR) aimed to gather studies describing the antibacterial action mechanisms and mode of trypsin inhibitors. The review protocol was registered (PROSPERO: CRD42020189069). Original articles resulting from studies in animal models, in bacterial culture, and using cells that describe antibacterial action of trypsin inhibitor-type peptides or proteins were selected in PubMed, Science Direct, Scopus, Web of Science, BVS, and EMBASE. The methodological quality assessment was performed using the PRISMA and OHAT tool. 2382 articles were retrieved, 17 of which were eligible. Four studies demonstrated the action mechanism directly on the bacterial membrane, and the fifth study on endogenous proteases extracted from the bacteria themselves. The antibacterial action mode was presented in the other studies, which can generate bacteriostatic or bactericidal effects without describing the mechanisms. This study generated information to enable new preclinical or clinical studies with molecules contributing to public health.

## Introduction

Diseases caused by infectious agents such as bacteria and viruses are among the leading causes of death and disability for thousands of people worldwide. They may have similar symptomatology; however, the therapeutic approaches are different. Bacterial infections are treated with antibiotics, which are inefficient in treating viral infections^1^. These diseases are characterised by histological, physiological, and biochemical injuries caused by the infectious agent^2^^,^^3^. When caused by resistant bacteria, the level of concern increases, as these bacteria are resistant to multiple drugs, require costly therapies, and are among the public health problems^2^^,^^4^.

Infectious diseases can be transmitted directly or indirectly by different routes: respiratory, faecal-oral, sexual transmission through objects, among others. Besides, it can be characterised by histological, physiological, and biochemical alterations of the lesions caused by the infectious agent^3^^,^^5^. These infections can affect the central nervous system, soft tissues of the head and neck, ocular regions, upper and lower respiratory tract, gastrointestinal tract, bone and joints, urinary tract, genital area, blood, and skin^6^.

One of the greatest concerns in the health area is the growing increase in infectious diseases capable of inducing death or disability in affected patients. Per World Health Organisation (WHO) statistics, in 2020, lower respiratory infections were among the ten leading causes of death, occupying the 4th position^7^. Over the past two decades, the world has seen six significant outbreaks of viral infectious agents (SARS-CoV: 2002–2004, Influenza H1N1: 2009–2010, MERS-CoV: 2012–2020, Ebola virus: 2013–2016, Zica virus: 2015 − 2016, Dengue and Chikungunya viruses and SARS-CoV-2: 2019-up to the present day). During the emergence of a viral disease, attention is focussed on treating the primary infection. Especially it is necessary to consider possible secondary opportunistic bacterial infections that may affect these patients^8^.

In parallel, this century is undergoing dramatic changes in the health needs of the world’s population. Although some countries project improvements in the global surveillance of infectious diseases, the clear majority of countries, especially low- and middle-income countries, remain vulnerable to outbreaks as they have fragile health systems^9^.

The knowledge of new products capable of acting against microorganisms deserves special attention today. Mortality rates from opportunistic infections due to resistance to antimicrobial drugs have increased^10^. In 2014, the WHO warned that the rapid emergence of resistant bacteria is a concern, and that the following are determining factors for the increase in resistance: (1) the extensive use and almost always unnecessary of antibiotics by the population, (2) the increased frequency of resistant phenotypes to these antimicrobials, (3) globalisation, which allows any pathogen to have access to humanity in different places due to greater geographical interconnection^11^^,^^12^.

The set of chemicals capable of fighting infectious diseases are known as antimicrobial agents and are effective in preventing, limiting, and eliminating the growth of microbial predators. Most of them were created from natural sources. This group includes agents or substances, such as antifungals, antiseptics, antibacterial, anthelmintics, antivirals, among others^12^.

Furthermore, the use of products obtained from plant sources with antimicrobial capacity has shown promise in the development of new drugs, as for the creation of active packaging capable of offering safer, better quality products with a longer shelf life. Besides, intelligent packaging can monitor the condition of the packaged food, providing information on its quality during transport and storage^13^^,^^14^.

Thus, the activities of bioactive molecules of plant origin have been widely studied, and among them, peptidase inhibitors and antimicrobial peptides (AMPs) stand out. Both are promising concerning antimicrobial activity and potential molecules for developing new biologically active products^13^. From this perspective, most studies are carried out *in vitro*, in animal models, or in cells models to understand the mechanisms of action before the clinical application of bioactive proteins or peptides.

Among the peptidase inhibitors, trypsin inhibitors have been evaluated in several studies. Some studies assess it in isolation, and others purified and characterised it extracted from different seeds. Trypsin inhibitor has been studied for its safety, biotechnological, and health application, evaluating the structural, chemical, and functional characteristics^15–18^. Thus, considering the multifunctionality of trypsin inhibitors, they are excellent candidates for studies to assess the antimicrobial potential and may behave as a protease inhibitor with AMP activity. However, its antibacterial action mechanisms or mode are unclear, unlike other bioactive compounds.

Given the highlighted points and considering the search for new alternatives for treating bacterial infections, the present study aimed to identify the mechanisms of antibacterial action of trypsin inhibitor-type peptides or proteins as a primary outcome through a systematic review (SR) study. For this, it was necessary to develop a SR protocol to analyse the original studies about the antibacterial action of trypsin inhibitor-type peptides or proteins to identify the mechanisms involved in the antibacterial action of these inhibitors.

## Methodology

This SR was prepared following the methodological criteria established by the Preferred Reporting Items for Systematic Reviews and Meta-analysis (PRISMA)^19^. The protocol for the construction of the review was registered in the International Prospective Register of Ongoing Systematic Reviews (PROSPERO), under number: CRD42020189069, and was based on the protocol described by Nascimento et al.^20^ Two independent investigators participated directly in all phases of the SR, with discrepancies resolved through discussion with a third reviewer^20^.

In this protocol, the scope of the review was registered, which included *in vitro* studies (bacterial culture), *in vivo* (rats and mice), and using cells, in which trypsin inhibitor-type peptides were administered to discover the mechanisms involved in the antibacterial action of these inhibitors. Primary endpoints were considered a mechanism of action, and secondary endpoints were a mode of action antibacterial of trypsin inhibitor-type peptides or proteins. It is noteworthy that antimicrobials can be classified per the mechanism of action, or even as to the mode of action or effect on microorganisms [classification based on pharmacodynamic parameters such as minimum inhibitory concentration (MIC) and minimum bactericidal concentration (MBC)]^21^.

### Search strategy

Searches for articles were performed using electronic searches in January 2021 in the following databases: PubMed, Science Direct, Scopus, Web of Science, Virtual Health Library (VHL), and EMBASE. Manual searches were performed based on search strategies established (Table 1). Searches in the databases were performed on a computer with the Federal University of Rio Grande do Norte (UFRN) IP, which allows access to all articles in each indexed database through the “Portal of Capes Periodicals” from Brazil.

**Table 1.** Search strategy equations for searching articles in databases that answer the question: what are the antibacterial mechanisms of action of trypsin inhibitor-type peptides or proteins?

Data base	Search equation
PubMed	Mechanism action AND peptide antibacterial AND trypsin inhibitor AND bacterial AND in vivo AND in vitro
SCIENCE DIRECT Used filter: Research articles	(Mechanism action) AND peptide antibacterial AND trypsin inhibitor AND in vivo AND in vitro
Scopus	Trypsin Inhibitor AND mechanism Antibacterial
EMBASE	('mechanism'/exp OR mechanism) AND ('trypsin inhibitor'/exp OR 'trypsin inhibitor') AND ('peptide antimicrobial' OR ('peptide'/exp OR peptide) AND ('antimicrobial'/exp OR antimicrobial) AND ('antibacterial activity'/exp OR 'antibacterial activity')
Web of Science	Mechanism AND trypsin inhibitor AND peptide antimicrobial AND bacterial
Virtual Health Library (VHL)	Mechanism antibacterial AND trypsin inhibitor AND bacterial AND *in vivo* AND *in vitro*

### Inclusion and exclusion criteria

#### Inclusion criteria

This SR included original experimental studies with rats and/or mice of both sexes, without water or diet restriction and original *in vitro* studies (bacterial culture) and original in cell studies, treated with peptides or proteins trypsin inhibitor type.

#### Exclusion criteria

Case studies without a control group, experiments with other types of animals, and studies that did not describe antibacterial action mode were excluded. Non-scientific articles were also excluded.

### Data extraction process

The selection of articles was initially performed by reading the title, abstract, and keywords without limitation of publication date using the Rayyan QCR web application^22^. Then, the complete reading was performed to analyse the inclusion and inclusion criteria exclusion defined.

The complete reading of the text was performed for data extraction: in animal studies (peptide used, bacterial strains used, MIC, positive control, mechanisms of antibacterial action), *in vitro* studies (peptide used, MIC, bacterial strains used, positive control, and mechanisms involved in antibacterial action), in cell studies (peptide used, MIC, positive control, cell type, bacterial strains used, and mechanisms of antibacterial action).

Data were grouped considering primary outcomes as antibacterial action mechanism and secondary outcomes as action mode of trypsin inhibitor type peptides or proteins.

### Risk of bias and quality evaluation

Two researchers were responsible for the independent readings. Discrepancies were resolved with the support of a third researcher. The reviewers were previously trained and calibrated to ensure uniformity in evaluating the criteria and Cohen’s kappa coefficient with a mean of 0.85. After reading the articles included in the review, a qualitative methodological assessment was conducted using the Office of Health Assessment and Translation (OHAT)^23^. The tool has eleven questions adaptable to each type of study. It can be used for *in vitro*, *in vivo*, and in cell studies with items scored as “definitely a low risk of bias,” “probably a low risk of bias,” “probably a high risk of bias,” “not reported,” (NR) “definitely a high risk of bias.” The specifications for evaluating each OHAT item for this review are described in protocol^20^.

## Result and discussion

### Selection and characteristics the studies

It is important to highlight that several search strategies were evaluated to answer the starting question efficiently. Thus, comparing the results obtained in each base, it was noticed that the strategies used were efficient.

Concerning the specific search strategies for each database, 2377 articles were found, and five articles were included through manual search. Of the total number of articles found, 56 were duplicates remaining 2326 for analysis. Initially, 1632 articles were identified in PUBMED, 26 articles in SCOPUS, 6 articles in the Web of Science, 10 articles in the VHL, 10 articles in EMBASE, and 693 articles in the Science Direct database. It is worth mentioning that, in the last cited database, there were 2006 articles. Still, the research articles filter was used, allowing the exclusion of review articles, conference abstracts, book chapters, editorials, short communications, and mini editorials. Subsequently, through manual search, five papers were added, totalling 2326.

**Figure 1.**
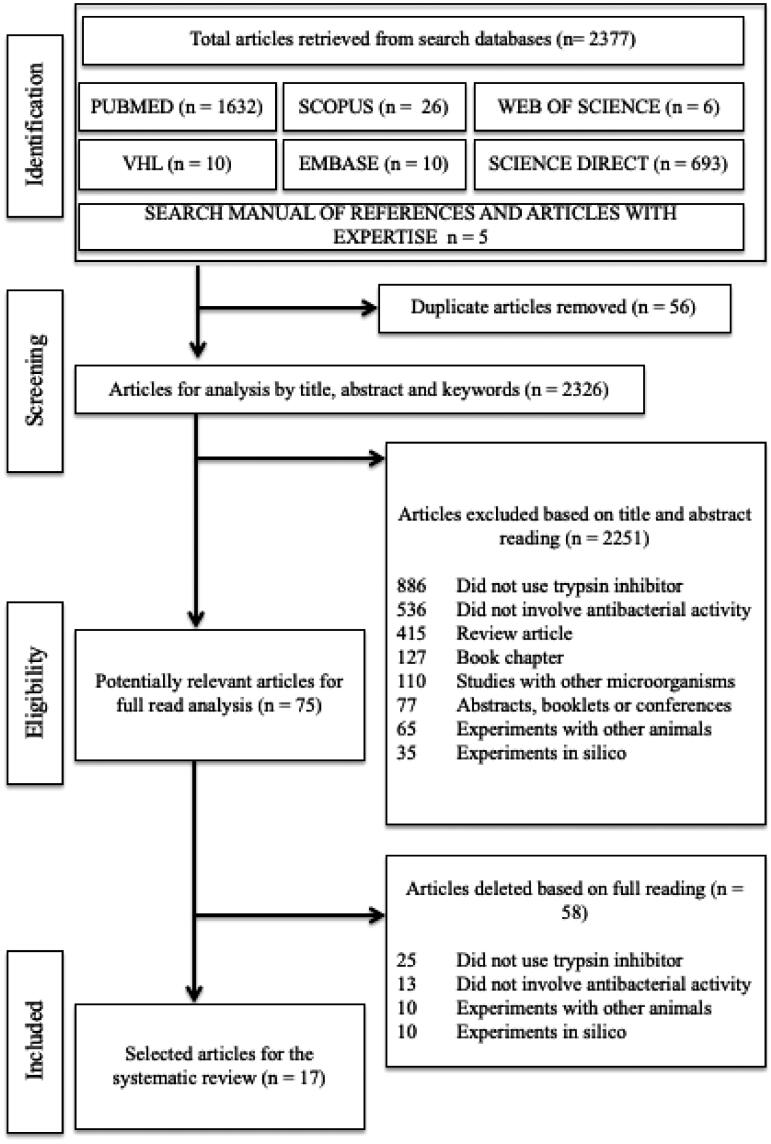
Flowchart for selecting articles per the Preferred Reporting Items Checklist for Systematic Review and Meta-Analysis (PRISMA) to answer the question: what are the antibacterial action mechanisms of trypsin inhibitor-type peptides or proteins?

After screening the title, abstract, and keywords, 75 articles were selected for screening the complete text. Among the excluded articles, it was observed that most (886) did not use trypsin inhibitors or did not involve antibacterial activity (536). Other studies were excluded because they used other animals (65), other microorganisms (110), or were focussed on *in silico* analyses (35). Finally, review articles (415), book chapters (127), and publications referring to abstracts, booklets, or conferences (77) were also excluded. At the end of the search, 17 were eligible for inclusion in the SR (Figure 1).

### Risk of bias and quality assessment

The studies’ bias risk and methodological quality were assessed in the seventeen eligible articles (Table 2). A higher risk of bias was perceived regarding items 1 and 2 related to dose administration and group allocation. The authors did not report random administration or random distribution of participants using standardised methods.

**Table 2.** Risk of bias and methodological quality assessment using the Office of Health Assessment and Translation (OHAT) tool to answer the question: what are the antibacterial mechanisms of action of trypsin inhibitor-type peptides or proteins?

References	P1	P2	P5	P6	P7	P8	P9	P10	P11
*In vitro* articles									
Malik et al. ^24^	DH	PL	DL	NR	PL	DL	DL	PL	NA
Rodrigues et al.^25^	DH	PH	DL	NR	PL	DL	DL	DL	NA
Martins et al. [26]	DH	PH	DL	NR	DL	DL	DL	DL	NA
Almeida et al. ^27^	DH	PH	DL	NR	DL	PL	DL	DL	NA
Yusoff et al. ^28^	DH	PH	PL	NR	PL	PL	PL	DL	NA
Wang et al. ^29^	DH	DH	DL	NR	DL	DL	DL	DL	NA
Costa et al. ^30^	PL	PL	DL	NR	PL	DL	DL	PL	NA
Szalapata et al. ^31^	DH	NR	DL	NR	PL	DL	DL	PL	NA
Chen et al. ^32^	DH	NR	DL	NR	PL	DL	DL	PL	NA
Liu et al. ^33^	DH	NR	PL	NR	DL	DL	PL	PL	NA
Yu et al. ^34^	DH	NR	DL	NR	PL	DL	DL	DL	NA
Li et al. ^35^	DH	NR	PL	NR	PL	PL	DL	PL	NA
Dabhade et al. ^36^	DH	NR	PL	NR	DL	DL	DL	DL	NA
Bacha et al. ^37^	DH	NR	DL	NR	PL	PL	DL	DL	NA
Bezerra et al. ^38^	DH	PL	DL	NR	DH	PL	PL	DH	NA
Mehmood et al. ^39^	DH	NR	DL	NR	DL	DL	DL	DL	NA
*In vivo* articles									
Malik et al. ^24^	DH	NR	DL	NR	PL	DL	DL	NR	NA
Wang et al. ^29^	PL	PL	DL	NR	PL	DL	DL	DL	NA
In cell articles									
Kaner et al. ^40^	DH	PL	DL	NR	PL	DL	DL	PL	NA

DL: definitely a low risk of bias; PL: probably a low risk of bias; PH: probably a high risk of bias; DH: definitely a high risk of bias; NR: not reported; NA: not applicable

Items 3 and 4 do not apply to *in vitro* and animal studies, so they were excluded. As for item 5, all articles had a good classification, with similar conditions for all groups.

Item 6, referring to the blinding of researchers, was highlighted in full with the option NR since any of the authors did not mention this information. Items 7–10 regarding presentation and reliability of results received good ratings for most studies. Finally, item 11, which refers to possible threats or internal validation, did not apply to any articles.

The score assigned to the studies was associated with a high risk of bias or unreported information on many issues. The articles did not contain enough detail to point out the risk of bias correctly. It is important to highlight that those experimental studies differ from randomised clinical trials, and random allocation to experimental and control groups is not standard practice in all experiments. Furthermore, the sample size in these studies is almost always relatively small^41^^,^^42^, which may justify assessments regarding the high risk of bias.

The disease (bacterial infection) was induced in most preclinical studies that evaluated the antibacterial action mode of peptides or trypsin inhibitors^24^^,^^29^, and posteriorly the trypsin inhibitor or its derivative was administered to assess the effect. Therefore, to reduce the risk of low scores, disease induction time should be the same for all groups, and it is essential to mention this information in the methodology. However, it was not available for some of the studies evaluated on this SR.

Finally, more information is needed concerning conditions under which these groups were maintained, allocated, and when the results were evaluated. Therefore, the need for greater clarity and detail of the methodological procedures adopted in the experiments was evidenced so that it is possible to improve the internal validation of the studies.

Although all peptides and proteins were trypsin inhibitors, they have different amino acid sequences. Adepamycin and Adevonin were the only trypsin inhibitors similar but still distinct. Furthermore, the studies were carried out with different bacteria, which revealed great heterogeneity, and different dosages of trypsin inhibitors were evaluated. Thus, it was not possible to carry out the intended meta-analysis.

The articles selected for SR were ordered by type of study *in vitro*, in animals, and using cells. The primary extracted data is presented in a compendium, facilitating understanding (Table 3). Among them: reference, the peptide used, bacteria, MIC, and mechanism of action. As well as secondary data (Table 4), among them a reference, the peptide used, bacteria, MIC, and action mode. Other data will be discussed throughout the text.

**Table 3.** Antibacterial action mechanisms of trypsin inhibitor-type peptides or proteins to answer the question: what are the antibacterial action mechanisms of trypsin inhibitor-type peptides or proteins?

References	Peptide	Bacteria	Cell type	Type of animal	Minimum inhibitory concentration (MCI)	Positive control	Mechanism of action
*In vitro* studies							
Martins et al.^26^	Lza BBI	*S. aureus*	NA	NA	5.8 × 10 ^−4 ^μM	Formaldehyde	The trypsin inhibitor was able to damage the membrane leading to cell lysis and promoted an increase in the production of reactive oxygen species (ROS)
Almeida et al.^27^	Adepamycin	*E. coli*	NA	NA	0.9 μM 3.6 μM	Chloramphenicol	The trypsin inhibitor was able to compromise the integrity of the membrane leading to the release of nucleic acids
Costa et al.^30^	JcTI-I	*S. enterica* *S. aureus*	NA	NA	0.5 μg/mL	–	The trypsin inhibitor was able to interact against bacterial proteases
Li et al.^35^	ORB1	*S. aureus*	NA	NA	1.76 μg/mL	–	The trypsin inhibitor was able to interact and break the cytoplasmic membrane, causing cell death

LzaBBI: synthetic inhibitor produced from the inhibitor of *L. auriculata* seeds; Adepamycin: synthetic inhibitor produced from Adenanthera pavonin trypsin inhibitor; JcTI-I: inhibitor produced from Jatropha curcas seed; ORB1: trypsin inhibitor produced from amphibian skin; NA: not applicable

**Table 4.** Antibacterial action mode of trypsin inhibitor-type peptides or proteins to answer the question: what are the antibacterial action mechanisms of trypsin inhibitor-type peptides or proteins?

References	Peptide	Bacteria	Cell type	Type of animal	Minimum inhibitory concentration (MCI)	Positive control	Mechanism of action
***In vitro* studies**							
Malik et al.^24^	pYR	*S. aureus* (ATCC 29213)	NA	NA	50 μM	Ampicillin	Bacteriostatic activity
Rodrigues et al.^25^	Adevonin	*K. oxytoca* (ATCC 13182) *S. aureus* (ATCC 80958)	NA	NA	1.84 µL 7.35 µL	Chloramphenicol	Bactericidal activity
Martins et al.^26^	Lza BBI	*S. aureus*	NA	NA	23.1 × 10 ^−4 ^μM	Formaldehyde	Bacteriostatic activity
Almeida et al.^27^	Adepamycin	*E. coli*	NA	NA	0.9 μM 3.6 μM	Chloramphenicol	Bacteriostatic activity
Yusoff et al.^28^	SMTI	*B. cereaus* *Erwinia* *Ralstonia* *S. typhi* *E. coli*	NA	NA	0.06 mg/mL 0.06 mg/mL 0.0015 mg/mL 0.12 mg/mL 0.48 mg/mL	–	Bacteriostatic activity
Wang et al.^29^	RV3	*E. coli* 25922 *E. coli* UB1005 *E. coli* K88 *E. coli* (K99) *S. pullorum* (7913) *S. typhimurium* (C7731) *S. typhimurium* (C14208) *P. aeruginosa* (27853) *S. choleraesuis* (CVCC503) *S. aureus* (29213) *S. aureus* (25923) *S. aureus* resistente à meticilina (MRSA 43300) *S. epidermidis* (12228) *E. faecalis* (29212)	NA	NA	2 μM 4 μM 1 μM 2 μM 1 μM 4 μM 1 μM 2 μM 1 μM 2 μM 2 μM 4 μM 2 μM 2 μM 2 μM 4 μM 1 μM 2 μM 2 μM 4 μM 4 μM 8 μM 2 μM 8 μM 2 μM 4 μM 2 μM 8 μM		Bacteriostatic and bactericidal activity
Costa et al.^30^	JcTI-I	*S. enterica* *S. aureus*	NA	NA	5 μg/mL	–	Bacteriostatic activity
Szałapata et al.^31^	AEBSF	*P. aeruginosa* *E. coli* *S. aureus*	NA	NA	0.5 mg/mL 3 mg/mL 2 mg/mL 4 mg/mL 3 mg/mL 3 mg/mL	–	Bacteriostatic and bactericidal activity
Chen et al.^32^	K-SL	*S. aureus*	NA	NA	64 μM	–	Bacteriostatic activity
Liu et al.^33^	PtPLC	*V. alginolyticus* *P. aeruginosa*	NA	NA	9.11 μM	–	Bacteriostatic activity
Yu et al.^34^	TIH3F	*E. coli (ATCC25922) E. coli (08040726)* *E. coli (08032813)* *E. coli (08032823)* *E. coli (08040726)* *S. dysenteriae (080420) K. Pneumoniae (08040202)* *K. Pneumoniae (08031012)* *P. mirabilis (1376)* *S. Maltophilia (090223)* *P. aeruginosa (08021015)* *S. aureus (08032706)* *S. aureus (08032810)* *S. aureus (08032615)* *B. subtilis (08042313)* *E. faecium (08052315) N. asteroides (08052412)* *S/ epidermidis*	NA	NA	9.38 μg/mL 4.69 μg/mL 75 μg/mL 9.38 μg/mL 75 μg/mL 4.69 μg/mL 75 μg/mL 75 μg/mL 9.38 μg/mL 9.38 μg/mL 9.38 μg/mL 75 μg/mL 75 μg/mL 2.37 μg/mL 4.69 μg/mL 4.69 μg/mL 2.37 μg/mL 37.5 μg/mL	Ampicillin	Bacteriostatic activity
Li et al.^35^	ORB1	*S. aureus*	NA	NA	1.76 μg/mL	–	Bacteriostatic activity
Dabhade et al.^36^	API	*P. aeruginosa MTCC 7926* *B. subtilis MTCC 1789*	NA	NA	2 μg/mL^−1^ 16 μg/mL^−1^	–	Bacteriostatic activity
Bacha et al.^37^	RfIP1	*B. cereus (ATCC 14579)* *B. subtilis (ATCC 6633)* *E. faecalis (ATCC 29122)* *S. epidermidis (ATCC 14990) S. aureus (ATCC 25923)* *E. coli (ATCC 25966)* *K. pneumonia (ATCC 700603)* *P. aeruginosa (ATCC 27853) S. entérica (ATCC 43972)*	NA	NA	2 μg/mL 3 μg/mL 3 μg/mL 5 μg/mL 4 μg/mL 11 μg/mL 10 μg/mL 13 μg/mL 14 μg/mL	Ampicillin	Bacteriostatic activity
Bezerra et al.^38^	IVTI	*E. Coli (ATCC 8739)*	NA	NA	25 μM	Chloramphenicol	Bacteriostatic activity
Mehmood et al.^39^	AnTI	*B. subtilis* *E. coli* *P. aeruginosa* *S. aureus* *X. oryzae*	NA	NA	20 μg/mL	Calamox	Bacteriostatic activity
*In vivo* studies (female mice)							
Malik et al.^24^	pYR	*S. aureus (*ATCC 29213)	NA	Female mice C57BL6	3.0 mg · kg^−1^	Neomycin	Bacteriostatic activity
Wang et al.^29^	RV3	*P. aeruginosa*	NA	Female BALB mice	8 µL	Ciprofloxacin	Bacteriostatic activity
In cell studies							
Kaner et al.^40^	(S-NO-hAAT)	*S. typhiforam*	Células de monócitos humanos, THP-1	NA	27 μM	Gentamicin	Bacteriostatic activity

pYR: peptide synthesised from anuran skin secretions; Adevonin: synthetic inhibitor produced from Adenanthera pavonin trypsin inhibitor; SMTI: trypsin inhibitor isolated from Streptomyces misionensis; LzaBBI: synthetic inhibitor produced from the inhibitor of *L. auriculata* seeds; Adepamycin: synthetic inhibitor produced from Adenanthera pavonin trypsin inhibitor; SMTI: trypsin inhibitor isolated from Streptomyces misionensis; RV3: peptide synthesised from sunflower trypsin inhibitor; JcTI-I: inhibitor produced from Jatropha curcas seed. AEBSF: 4–(2-aminoethyl) benzenesulfonyl serine protease inhibitor; K-SL: inhibitor synthesised from frog skin secretion; PtPLC: inhibitor synthesised from the arthropod serine protease inhibitor, *Portunus trituberculatus*; TIH3F: peptide synthesised from the junction of cathelicidin with a trypsin inhibitory loop; ORB1: trypsin inhibitor produced from amphibian skin; API: trypsin inhibitor from Albizia amara seeds; RfIP1: Rhamnus frangula trypsin inhibitor; IVTI: Inga Vera seed trypsin inhibitor; AnTI: Acacia nilotic L trypsin inhibitor; S-NO-hAAT: inhibitor synthesised from human α1-antitrypsin; NA: not applicable

### Antibacterial action mechanisms

AMPs can be found in animal and plant species, and there is great interest in exploring the potential of these peptides as bactericidal agents. The ability of AMPs to neutralise endotoxemia/sepsis and stimulate innate host responses while attenuating potentially deleterious inflammatory responses makes them stand out over other bactericidal agents^24^^,^^43^^.^ Thus, there is great interest in exploring the potential of these peptides.

Furthermore, antimicrobials can be grouped in various ways. Classically, they can be grouped according to the spectrum of action, the chemical structure, and the effect on microorganisms^44^. Antimicrobial agents exert their mechanisms of action in various ways and can interfere with cell wall synthesis, altering the altering the cytoplasmic membrane's permeability, promoting protein synthesis changes, inhibiting nucleic acid synthesis, and interfering with chromosome replication^21^^,^^44^. Thus, the bacterial membrane is an important target for many antibacterial formulations^45^^,^^46^.

In this review, four studies^26^^,^^27^^,^^30^^,^^35^ investigated and presented the action mechanisms of trypsin inhibitors on the bacterial membrane. Martins et al.^26^ evaluated *in vitro* the action mechanism of the Bowman-Birk serine protease inhibitor from *Luetzelburgia* *auriculata* (Lza BBI) seeds on Staphylococcus *aureus*. They observed the impact of a concentration of 5.8 × 10^−4 ^μM on the bacterial membrane by scanning microscopy. Thus, they concluded that the antibacterial effect of Lza BBI is mainly due to oxidative stress promoted by the production of reactive oxygen species (ROS) and damage to the cell membrane that leads to cell lysis.

Li et al.^35^ investigated, in another *in vitro* assay, the action of a trypsin inhibitor produced from amphibian skin, called ORB1, on *S. aureus* (ATCC 2592). They observed by scanning microscopy that the inhibitor promoted bacterial death, directly affecting its cell wall and membrane. Large laminar mesosomes emerged from the septa and cell wall of bacteria treated with ORB1. On the other hand, no mesosome structure was detected in the control group of untreated bacteria^35^.

From the results of this study, the interface between cell wall and membrane is unclear. In some regions, the interface has disappeared due to the lysis of both or their separation. Therefore, the authors suggest that ORB1 interacted and ruptured the cytoplasmic membrane, leading to its dissolution and finally to the death of the cell itself. This process shows how the putative mode of cationic AMPs acts on Gram-positive bacteria^35^.

Using a peptide developed from the *Adenanthera pavonina* trypsin inhibitor (ApTI) sequence, called Adepamycin, also in an *in vitro* study, Almeida et al.^27^ pointed out the mechanism of action of ApTI on the *Escherichia coli* membrane. From a nucleic acid release assay (DNA and RNA), it was possible to observe that the content of free nucleic acids increased 7-fold in bacteria incubated with Adepamycin, demonstrating that Adepamycin could compromise the integrity of the membrane, leading to the release of nucleic acids.

It is known that selective permeability is an essential feature of the cytoplasmic membrane, which regulates the passage of substances into cells and the output of waste from cell catabolism^47^. Bacterial death can occur when there are physical-chemical changes in the cytoplasmic membrane. This change can lead to the exit of essential substances from the cell, such as phosphates and nucleic acids, and facilitate the entry of harmful elements to bacterial cell metabolism^21^^,^^47^.

Additionally, Malanovic and Lohner^48^ described that for a peptide to break the membrane of a Gram-positive bacterium, such as *S. aureus*. Initially, it accumulates on the membrane surface until reaches a critical concentration, as these peptides need to diffuse through the pores. These data corroborate Lee et al.^49^, which reinforce the need for these peptides to first diffuse into the peptidoglycan matrix and then over the cytoplasmic membrane.

Finally, Costa et al.^30^ examined *in vitro* the action of a peptide extracted from the seed of *Jatropha curcas*, JcTI-I, on endogenous proteases extracted from the bacteria themselves, *Salmonella enterica and S. aureus*. They observed that the peptide caused 100.0% inhibition of proteases extracted from *S. enterica* and almost 84.6% of those extracted from *S. aureus*. Therefore, raising another mechanism that trypsin inhibitors can use to confer antibacterial activity.

Based on the information provided by these studies and knowing the peptide sequences of these inhibitors tested in the different models evaluated, it is possible to elucidate the interactions between the inhibitors and the lipid membranes of bacteria. Thus, characteristics such as hydrophobicity, amphipathicity, presence of cationic residues (in particular arginine/Arg or lysine/Lys), size, and structural conformation of these peptides are correlated with the access of these molecules through the lipid bilayer of biological membranes. Besides, they are associated with the consequences for the cells involved^43^. Undoubtedly, this knowledge can be applied to improve the therapeutic action of these molecules as part of the global challenge to overcome antimicrobial resistance.

In one of the studies evaluated in this SR, for the creation of the rational Adevonine design, a synthetic peptide produced from the trypsin inhibitor of ApTI, Rodrigues et al.^25^ selected a sequence of amino acid residues containing tyrosine (Tir), phenylalanine (Phe), and Arg, which added up to a ^+2^ charge. To make Adevonine more cationic, the authors changed the amino acid composition by adding more Arg and Lys residues and changing the charge to ^+6.^ These changes gave Adevonine a greater antibacterial activity when compared to the original inhibitor, ApTI.

Almeida et al.^27^ also synthesised Adepamycin, a peptide designed from part of the amino acid sequence of the trypsin inhibitor, ApTI. The authors substituted seven amino acid residues, including glycine (Gly), serine (Ser), Gly, and Proline (Pro) residues for histidine (His), alanine (Ala), Arg, and Lys, in that order. In the same way as Rodrigues et al.^25^, they also observed an increase in the net positive charge, from ^+3^ to ^+6^, replacing amino acids. Concerning antibacterial activity, the authors found that, unlike the parent peptide of ApTI, which did not show antibacterial activity at the maximum concentration tested (10 μM), Adepamycin showed bacteriostatic (0.9 μM) and bactericidal (3, 6 μM), against *E. coli*.

Also, in the study mentioned above, Almeida et al.^27^ pointed out that, in atomic terms, the amino acids phenylalanine (Phe), glutamine (Gln), arginine (Arg), and histidine (His) were the primary amino acid residues interacting with the phospholipids in the bilayer complex (in the ratios 3:1 w/w of 1-palmitoyl-2-oleoyl-sn-glycerol-3-phosphoglycerol) at the end of the simulations. In addition, Arg and His residues were involved in 16 of the 21 interactions performed with the bilayer/Adepamycin complex, revealing the importance of these basic polar residues for the action of Adepamycin on bacterial surfaces^27^.

Although several articles were found involving the antibacterial action mode of trypsin inhibitors, those describing the action mechanism in detail are limited. Furthermore, the detailed mechanism showing how these peptides penetrate through bacterial barriers depends on some changes, which have not been described. Therefore, it is a limitation of the evaluated studies. In Gram-positive bacteria, peptides must first diffuse through the peptidoglycan matrix and then over the cytoplasmic membrane. In contrast, toxicity involves a disturbance or rupture of the cytoplasmic membrane and the outer membrane in Gram-negative bacteria. On the other hand, the inability to permeabilize or rupture the outer membrane results in the absence of antimicrobial activity.^49^ Above all, a large part of the articles presented their action mode, even not showing the mechanisms of action against various Gram-positive and Gram-negative bacteria, also directing the gaze towards only the effect.

### Antibacterial action mode

The improvement of infections is directly related to the action of agents capable of influencing bacterial growth or altering bacterial viability. Based on their mode of action on bacterial cells, antibacterial are divided into two groups: bactericides or bacteriostatics^50^. Bactericides kill bacteria, and bacteriostatic suppress bacterial growth (keep them in the stationary phase of growth). Therefore, it is observed from the gathered findings that trypsin inhibitors can generate both bacteriostatic and bactericidal effects. Such classification is made per pharmacodynamic parameters, such as the MIC and the MBC^50^.

Malik et al.^24^ tested the action of pYR, a peptide produced from anuran skin secretions *in vitro* and *in vivo* against *S. aureus*. This peptide was submitted to a cyclisation process, promoting increased stability but reduced bioactivity. Thus, this can negatively interfere with antimicrobial activity. The peptide did not cause toxicity in the dosage used. In both cases (*in vitro* and *in vivo*), there was a reduction in bacterial growth/load, being characterised as a bacteriostatic effect. The authors reinforce that the relationship between antimicrobial agents' *in vitro* and in vivo efficacy is not completely clear and predictable. Besides, the in vivo action may be influenced by parameters not controlled in the host.

Wang et al.^29^ also, investigated the action of a peptide, RV3 produced from sunflower trypsin inhibitor, through *in vitro* and *in vivo* studies against several bacteria: *E. coli* (25922), *E. coli* (UB1005), *E. coli* (K88), *E. coli* (K99), *Salmonella pullorum* (7913), *S. typhimurium* (C7731), *S. typhimurium* (C14208), *Pseudomonas aeruginosa* (27853), *S. choleraesuis* (CVCC503), *S. aureus* (29213), *S. aureus* (25923), *methicillin-resistant S. aureus* (MRSA 43300), *Staphylococcus epidermidis* (12228), *Enterococcus faecalis* (29212). RV3 showed bacteriostatic and bactericidal effects. The authors also investigated the action of this peptide *in vivo* in a model of infection (dermatitis caused by *P. aeruginosa* 27853) in the mice’s skin. They found that RV3 inhibited the infiltration of inflammatory cells and promoted the proliferation of cells in the hair follicle protecting the epidermal cells.

Kaner et al.^40^ evaluated the effect of the S-NO-hAAT peptide, synthesised from the human α1-antitrypsin inhibitor, on immune system cells (human monocytes, THP-1) during a bacterial infection caused by *Salmonella typhi*. E and verified bacteriostatic effect at the concentration of 27.5 µM, macrophages activated by S-NO-hAAT were responsible for the death of bacteria in the intracellular environment. The authors considered the possibility that some S-NO-hAAT activities may involve transnitrosylation, that is, the transfer of NO molecules of this peptide to cellular targets. Therefore the peptide may exert an anti- or pro-inflammatory effect, suggesting further studies with different cell lines.

Yusoff et al.^28^ also observed a bacteriostatic effect, using the SMTI peptide, synthesised from the local strain of actinomycetes (Kuala Lumpur, Malaysia) against *Bacillus cereus*, *Erwinia, Ralstonia*, *S. Typhi*, and *E. coli*, using varying CIM from 0.0015 to 0.48 mg/mL.

On the other hand, Mehmood et al.^39^ identified a bacteriostatic effect using 20 μg of AnTI, a trypsin inhibitor of *Acacia nilotica* L., on *Bacillus subtilis, E. coli, P. aeruginosa, S. aureus,* and *X. oryzae.*

Using much lower inhibitory concentrations, ranging from 2 to 14 μg/mL, Bacha et al.^37^ observed a bacteriostatic effect of a *Rhamnus frangula* trypsin inhibitor, RfIP1 against several Gram-positive and Gram-negative bacteria, *B. cereus* (ATCC 14579), *B. subtilis* (ATCC 6633), *E. faecalis* (ATCC 29122), *S. epidermidis* (ATCC 14990), *S. aureus* (ATCC 25923), *E. coli* (ATCC 25966), *K. pneumonia* (ATCC 700603), *P. aeruginosa* (ATCC 27853) and *S. enterica* (ATCC 43972).

Dabhade et al.^36^ evaluated the effect of API, a trypsin inhibitor from *Albizia amara* seeds on *P. aeruginosa* (MTCC 7926) and *B. subtilis* (MTCC 1789). They identified a bacteriostatic effect on MIC of 2 μg/mL and 16 μg/mL, respectively.

Rodrigues et al.^25^ investigated the *in vitro* action of Adevonin, a peptide developed from the sequence of the ApTI, on the membranes of *Klebsiella oxytoca* (ATCC 13182) and *S. aureus* (ATCC 80958). The results showed a bactericidal effect, unlike the antibiotic chloramphenicol used as a control, which caused a bacteriostatic action. This study also observed the influence of Adevonin on other bacteria, including Gram-positive and Gram-negative: *E. coli* (ATCC 35218), *Klebsiella pneumoniae* (ATCC 70603), *Serratia marcescens* (ATCC 13880), *K. pneumoniae* KpC + (001825971 isolated) with MIC of 7.35, 3.67, and 1.84 µL, respectively, thus resulting in a bacteriostatic effect.

In another study, with an inhibitor synthesised from ApTI entitled Adepamycin *in vitro*, Almeida et al.^27^ observed its bacteriostatic action against several bacteria, including Gram-positive *S. aureus* bacteria with a MIC of 1.8 µM, and Gram-negative: *K. pneumoniae, P. aeruginosa, K. oxytoca* with the following MICs: 1.4, 2.8, and 3.6 µM. In this study, the authors highlighted the action of ApTI on *E. coli*, which showed a bacteriostatic effect at 0.9 µM at MIC and a bactericidal effect at a concentration of 3.6 µM.

With a greater focus on *E. coli*, Yu et al.^34^ designed a peptide from the junction of cathelicidin with a trypsin inhibitory loop, called TIH3F. In this study, a bactericidal effect was observed only for *E. coli* (ATCC25922), using a concentration of 46.9 μg/mL. In the other bacteria, a bacteriostatic effect was observed in Gram-negative: *E. coli* (ATCC25922), *E. coli* (08040726), *E. coli* (08032813), *E. coli* (08032823), *E. coli* (08040726), *S. dysenteriae* (0804203), *K. pneumoniae* (08040202), *K. pneumoniae* (08031012), *Proteus mirabilis* (1376), *S. maltophilia* (090223), *P. aeruginosa* (08021015) with MIC ranging from 4.69 to 75 µg/mL, and also about Gram-positive: *S. aureus* (08032706*), S. aureus* (08032810), *S. aureus* (08032615), *B. subtilis* (08042313), *E. faecium* (08052315), *N. asteroides* (08052412), *S. epidermidis* with MIC ranging from 2.37 to 75 μg/mL.

One of the many factors to predict a favourable clinical outcome of the action potential of antimicrobial agents can be provided using bactericidal/bacteriostatic data by MIC. Therefore, MICs are used in clinical situations mainly to confirm resistance and determine the *in vitro* activities of new antimicrobials^50^.

Liu et al.^33^ tested *in vitro* the action of the PtPLC peptide synthesised from an arthropod serine protease inhibitor, *Portunus trituberculatus*, against *V. alginolyticus* and *P. aeruginosa*. In that study, it was identified that a MIC of 9.11 μM was sufficient to observe only a bacteriostatic effect.

Szalapata et al.^31^ identified a bacteriostatic effect of a synthetic inhibitor of the serine protease type, 4–(2-aminoethyl) benzenesulfonyl fluoride hydrochloride (AEBSF) on *P. aeruginosa, E. coli*, and *S. aureus* in the following MICs: 0.5, 2, and 2 mg/mL, respectively. As well as bactericidal effect at concentrations of 3, 4, and 3 mg/mL, respectively. It is noteworthy that AEBSF resulted in bacteriostatic and bactericidal effects against *S. aureus* using the same concentration.

With bacteriostatic effect, several studies have reported antibacterial action of trypsin inhibitor-type peptides or proteins. Bezerra et al.^38^ evaluated *in vitro* the effect of IVTI, a trypsin inhibitor from *Inga vera* seeds, and observed a bacteriostatic effect at 25 μM MIC on *E. coli* (ATCC 8739).

Chen et al.^32^ evaluated the antimicrobial activity of a synthetic trypsin inhibitor produced from the skin secretion of broad-folded frog (*Sylvirana latouchii*): K-SL, on *E. coli* (ATCC 11775), *S. aureus* (ATCC 12600), and methicillin-resistant *S. aureus* (MRSA) (NCTC 12493) observing bacteriostatic effect only against *S. aureus* at a concentration of 64 μM.

Meanwhile, Li et al.^35^ investigated the mode of action of a trypsin inhibitor produced from amphibian skin, called ORB1, on *S. aureus* (ATCC 2592), *E. coli, B. subtilis*, and their respective MICs: 1.76, 2.34, and 2.34 μg/mL observed a bacteriostatic effect.

Martins et al.^26^ investigated the effect of a Bowman-Birk serine protease inhibitor from *L. auriculata* (Lza BBI) seeds on *S. aureus* and identified an *in vitro* bacteriostatic effect at a concentration of 23.1 × 1 0 ^−4 ^μM. In addition to a bactericidal effect at a concentration of 92.5 × 1 0^−4 ^μM.

Among the studies presented in this review, *E. coli* and *S. aureus* were evaluated in more than one. Even in different studies, using inhibitors extracted from various sources and with varying units of measurement, it was possible to point out that those that involved trypsin inhibitors and *E. coli*, dosages sufficient to prevent bacterial growth ranged from 0.9 to 2.0 μM. And in studies involving trypsin inhibitors and *S. aureus*, enough dosages to inhibit bacterial growth ranged from 2.0 to 64.0 μM, up to now all *in vitro* studies. These findings and others will serve to guide the choice of dosages to be used in future research involving these and other trypsin and bacterial inhibitors.

**Figure 2.**
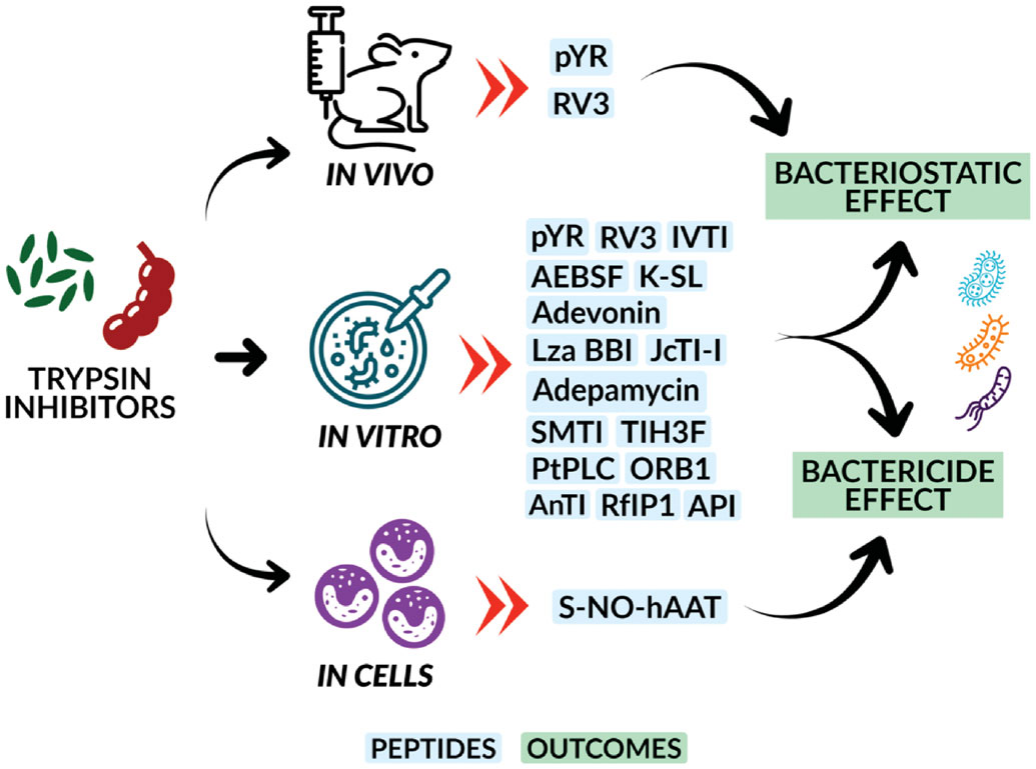
Antibacterial effects of trypsin inhibitors. Trypsin inhibitors can act by generating a bactericidal effect, causing the death of bacteria or a bacteriostatic effect, where bacterial growth is suppressed (keeping them in the stationary growth phase). pYR: peptide synthesised from anuran skin secretions; RV3: peptide synthesised from sunflower trypsin inhibitor; IVTI: Inga Vera seed trypsin inhibitor; AEBSF: 4-(2-aminoethyl) benzenesulfonyl serine protease inhibitor; K-SL: inhibitor synthesised from frog skin secretion; Adevonin: synthetic inhibitor produced from Adenanthera pavonin trypsin inhibitor; LzaBBI: synthetic inhibitor produced from the inhibitor of *L. auriculata* seeds; JcTI-I: inhibitor produced from Jatropha curcas seed; Adepamycin: synthetic inhibitor produced from Adenanthera pavonin trypsin inhibitor; SMTI: trypsin inhibitor isolated from Streptomyces misionensis; TIH3F: peptide synthesised from the junction of cathelicidin with a trypsin inhibitory loop; PtPLC: inhibitor synthesised from the arthropod serine protease inhibitor, *Portunus trituberculatus*; ORB1: trypsin inhibitor produced from amphibian skin; AnTI: Acacia nilotic L trypsin inhibitor; RfIP1: Rhamnus frangula trypsin inhibitor; API: trypsin inhibitor from Albizia amara seeds; S-NO-hAAT: inhibitor synthesised from human α1-antitrypsin

Finally, given the studies included in this SR, it was possible to verify that a large part of the trypsin inhibitors, whether from plants or animals, could generate bacteriostatic or bactericidal effects depending on the concentrations used (Figure 2). It was noticed that only three studies pointed to the likely mechanism that led to the antibacterial effect. Two studies showed that the action of the inhibitors was directly on the bacteria membrane, both against *S. aureus* Gram-positive bacteria, and the described mechanism given to the peptides caused membrane disturbance, generating cell lysis. One study presented an action mechanism against Gram-negative bacteria *E. coli*. The trypsin inhibitor could compromise the integrity of the membrane leading to the release of nucleic acids. In a fifth study, the effect was attributed to the antibacterial activity by the evaluated inhibitor, and it was the inhibition of the endogenous proteases extracted from the bacteria themselves.

However, for one of these trypsin inhibitor-type peptides or proteins to be used as a therapeutic agent or in the food industry, it must have properties such as (1) high antimicrobial activity, (2) low toxicity, (3) high proteolytic stability, and (4) low cost^51^^,^^52^. Thus, significant efforts are needed to design new peptides with high antimicrobial activity. Some technologies have been used to fill this gap, such as bioinformatics, based on more sophisticated mechanisms such as molecular dynamics simulations and biophysical experiments. Recently, approaches combining computational predictions, biophysical characterisations, and biological validations have shown promise^51^. From these methods related to in silico studies, it is possible to project changes in the conformation of peptides and observe the impact on the stability and bioactivity of these molecules before performing antimicrobial tests^24^. On the other hand, this SR did not include original articles with studies exclusively *in silico*, although this type of approach was also observed in some selected papers^24^^,^^25^^,^^27^^,^^32^^,^^34^.

Finally, considering the types of original studies included in this review, the present SR showed that trypsin inhibitors evaluated *in vitro*, *in vivo*, or in cell studies presented mechanisms capable of reducing or eliminating bacterial action. This evidence may improve the choice of trypsin inhibitors for possible applications seeking future treatments with specific and promising targets. Based on this, new studies (preclinical or clinical) using trypsin inhibitors may contribute to public health, assisting in maintaining populations' health, aiming at new promising treatments for bacterial infections.

## Conclusions

Trypsin inhibitors were evaluated for mechanisms of action to suppress bacterial infection. We can see that natural and synthetic compounds act in different ways to promote antibacterial action. Only four studies investigated the mechanism of action directly on the bacterial membrane. The authors pointed out membrane disturbances in three studies using *S. aureus* and *E. coli*. A fifth study evaluated the antibacterial effect on endogenous proteases extracted from the bacteria themselves (*S. enterica* and *S. aureus*). In other studies, the antibacterial action mode was presented, which can generate bacteriostatic or bactericidal effects depending on the concentrations used.
